# Colorless Semi-Alicyclic Copolyimides with High Thermal Stability and Solubility

**DOI:** 10.3390/polym11081319

**Published:** 2019-08-07

**Authors:** Zhongxu Lan, Chunyu Li, Yanlei Yu, Jia Wei

**Affiliations:** Department of Materials Science & State Key Laboratory of Molecular Engineering of Polymers, Fudan University, Shanghai 200433, China

**Keywords:** copolyimide, solubility, optical transparency, thermal stability

## Abstract

A series of colorless copolyimide films with high thermal stability and good solubility are synthesized from (trifluoromethyl)biphenyl-4,4’-diamine (TFMB) with different 1,2,4,5-cyclohexanetetracarboxylic dianhydride (HPMDA) to 2,2-bis(3,4-dicarboxyphenyl)-hexafluoropropane (6FDA) dianhydride mole ratios through one-pot solution polycondensation. These copolyimide films exhibit excellent optical transparency (*T*_400_ > 90% and *λ*_0_ ~305–333 nm) with a thickness of 15 μm and good solubility in most organic solvents. The excellent optical properties are mainly attributed to the low inter- and intra-molecular charge transfer interactions due to the alicyclic structure and the strong electronegative CF_3_ groups. The glass transition temperature increases from 332 to 352 °C with increasing HPMDA content in the copolymers, while the thermal decomposition temperature is improved with increasing 6FDA content. These results indicate that the copolyimide films can be successfully utilized in the development of novel heat-resistant plastic substrates for the optoelectronic engineering applications.

## 1. Introduction

Polyimide (PI) has been extensively applied in fields like aerospace, microelectronics and optoelectronics owning to its excellent combination properties, such as high thermal stability, low dielectric constant, excellent chemical resistance and mechanical properties [1,2,3]. Conventional aromatic polyimide films generally exhibit deep coloration generated by the formation of intra- and inter-molecular charge transfer complex (CTC) due to their highly conjugated molecular structures, which significantly limits their applications in which optical transparency is very important, such as optoelectronics devices [4,5,6]. Colorless polyimide (CPI) films have attracted great interest for their potential applications in optoelectronic devices, specifically in flexible display, flexible printing circuit boards and flexible thin film solar cells [7,8,9]. However, achieving a breakthrough in the molecular design of CPI, particularly with regard to the trade-off between the good transparency and thermal stability of PI films, remains challenging because of the contradiction between the two properties [7,8].

Recently, a considerable amount of research effort has been devoted for the improvement of PIs’ optical transparency through rational structural modification. Successful methodologies mainly include the incorporation of fluorine atoms [10,11,12,13,14,15], alicyclic moieties [16,17,18,19,20,21], bulky substituents [22,23,24,25,26,27] and asymmetric or rigid but non-coplanar segments [28,29,30,31]. These structural adjustments can hinder conjugation, loosen PI chain stacking, inhibit CTC formation, and thus obtain CPI [7,8,9]. Of the methods mentioned above, introducing fluorinated and alicyclic structure is two effective ways for synthesizing CPI [7,8]. Fluorinated aromatic PIs usually exhibit improved optical transparency and enhanced solubility because of the strong electronegativity and large free volume of the fluorinated groups [5,7,8]. Unfortunately, these fluorinated aromatic PIs still remain to have a pale color in the visible region attributed to the presence of charge transfer (CT) interactions caused by the aromatic backbones [5]. Alicyclic PIs always display higher transparency and better solubility compared with aromatic PIs, which is ascribed to their relatively low polarity, low molecular density and low probability of undergoing inter- and intra-molecular CT interactions [7]. Alicyclic PIs show higher transparency, but lower thermal decomposition temperature than fluorinated aromatic PIs, which restricts its application [7]. Thus, combining fluorinated aromatic monomers with alicyclic monomers is an effective way to achieve semi-alicyclic PIs with desired optical transparency, thermal stability and solubility.

However, the main problem in the fabrication of semi-alicyclic PIs is the low reactivity of most alicyclic monomers [7,8]. Generally, two-step thermal imidization is unsuitable for semi-alicyclic PI synthesis due to the low reactivity of alicyclic monomers, which results in the low molecular weight of the semi-alicyclic PI and brings difficulty for film-forming [32]. For this problem, one-pot solution polycondensation was developed [33]. The imidization in the one-pot PI synthesis is completed in the solution state and a relatively mild reaction temperature is used, which probably avoids side reactions in curing process at the high temperature solid phase [33,34]. In the general two-step thermal imidization process, PAA (poly(amic acid)) solution is synthesized first and then the curing process is completed in programmed heating. One practical issue is the PAA solution may degrade in long-term storage. However, one-pot solution polycondensation avoids the problem, because it uses soluble PI in the film-forming process instead of PAA solution.

Herein, an alicyclic monomer 1,2,4,5-cyclohexanetetracarboxylic (HPMDA), two fluorinated aromatic monomers, 2,2-bis(3,4-dicarboxyphenyl)hexafluoropropane dianhydride (6FDA) and 2,2’-bis(trifluoromethyl)benzidine (TFMB) were copolymerized for the fabrication of CPIs with high thermal stability and good solubility. One-pot solution polycondensation was used to synthesize CPIs with moderated molecular weight. The influence of mole ratio of HPMDA/6FDA on the solubility, thermal properties, optical performance and mechanical properties of the CPIs was investigated. The copolymerization of fluorine-contained and alicyclic monomers endows the CPIs with adjustable properties. Most of the obtained films possess excellent overall performances, which expand their applications in optoelectronic engineering.

## 2. Experimental

### 2.1. Materials

2,2-bis(3,4-dicarboxyphenyl)hexafluoropropane dianhydride (6FDA) and 2,2′-bis- (trifluoromethyl)biphenyl-4,4′-diamine (TFMB) and 1,2,4,5-cyclohexanetetracarboxylic dianhydride (HPMDA) were purchased from Shenzhen Feiming Science and Technology Co., Ltd (Shenzhen, China) and used as received. Anhydrous grade dimethylacetamide (DMAc), dimethylformamide (DMF), dimethyl sulfoxide (DMSO), tetrahydrofuran (THF), ethyl acetate (EA), acetone, dichloromethane (DCM), m-cresol, isoquinoline, toluene, methanol (MeOH) and ethanol were purchased from Adamas (Shanghai, China) and used without purification.

### 2.2. Preparation of Homopolyimide and Copolyimide Films

**Scheme 1:**
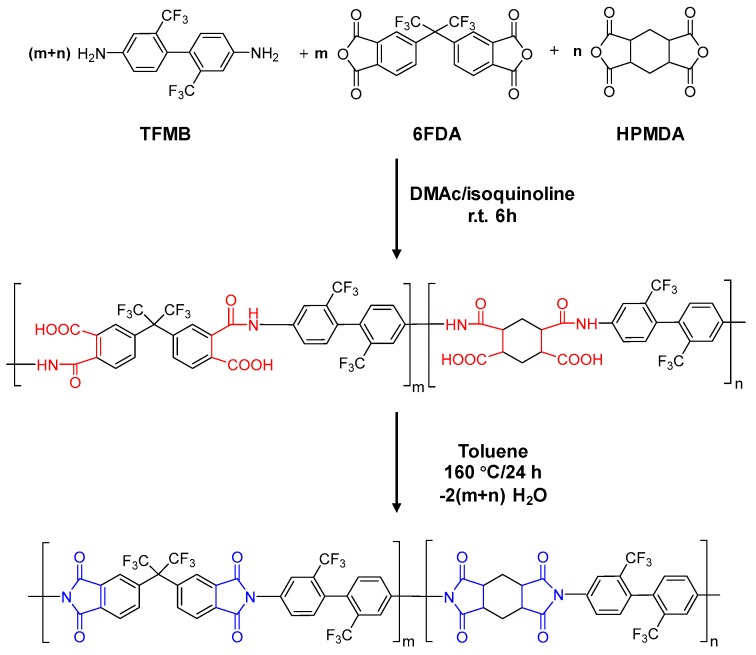
Synthesis of homopolyimides and copolyimides.

A series of homopolyimide and copolyimide films were synthesized from TFMB, 6FDA and HPMDA via one-pot solution polycondensation (Scheme 1). The feed mole ratios of HPMDA/6FDA in the copolyimides were 0:10, 1:9, 4:6, 6:4, 8:2 and 10:0, corresponding to PI-0, PI-10, PI-40, PI-60, PI-80 and PI-100. In a typical experiment, PI-80 was synthesized through the following procedures: TFMB (3.202 g, 10 mmol) was dissolved in anhydrous DMAc (10 mL) in a completely dried 100 mL three-necked flask equipped with a magnetic stirrer and a dean-stark trap under nitrogen. 6FDA (0.89 g, 2 mmol), HPMDA (1.794 g, 8 mmol) and isoquinoline (0.03 g) were then added and the mixture was stirred at room temperature under nitrogen flow for 6 h followed by adding 5 mL toluene into the mixture. The mixture was then gradually heated to 160 °C to induce imidization and stirred for 24 h. The water evolved in the procedure of imidization was removed simultaneously by azeotropic distillation. The mixture was cooled down to 80–100 °C, and then poured slowly into excess ethanol, forming precipitates, which were collected by filtration. The precipitates were washing thoroughly with ethanol followed by drying in vacuum oven at 100 °C for 12 h and PI powder was obtained. The PI powder was dissolved in DMAc at a solid content of 15 wt %. The solution was filtered by a Teflon syringe filter with 0.22 μm aperture and then spread onto glass substrates followed by baking to remove the solvent. The temperature was increased successively to 60, 80, 100 and 150 °C, and the sample was allowed to stand at each temperature for 1 h. The free-standing polyimide film was then immersed in deionized water to facilitate removal from the glass substrates and dried at 100 °C in a blast oven.

### 2.3. Characterization

FT-IR spectra were acquired using a Bruker VERTEX 70 spectrometer (Bruker, Karlsruhe, Germany) ranging from 400 to 4000 cm^−1^ at a resolution of 4 cm^−1^ using the transmission mode with a KBr pellet. The FT-IR spectra were conducted with a baseline correction. ^1^H NMR spectra were collected using a Bruker AVANCE III spectrometer (400 MHz, Bruker, Karlsruhe, Germany) in dimethylsulfoxide-*d*6. Gel permeation chromatography (GPC) was conducted on a Waters e2695 instrument (Waters, Milford, MA, USA) with THF as the eluent at flow rate of 1 mL/min at 50 °C. The solubility behavior was determined by dissolving the polyimides in different solvents with a concentration of 5 wt % at room temperature or at elevated temperatures. Differential scanning calorimetry (DSC) was performed on a TA instrument Q2000 (TA, Newcastle, DE, USA) and the procedure was listed as follows: Heating the sample to 400 °C at 20 °C/min in 50 mL/min nitrogen flow with about 4 mg samples (powder) and cooling down to 40 °C at 20 °C/min in 50 mL/min nitrogen flow, then reheating the sample to 400 °C at 10 °C/min in 50 mL/min nitrogen flow. The second heating cycle was used to determine the glass transition temperature (*T*_g_). Thermogravimetric analysis (TGA) was performed on a TA instrument Q500 (TA, Newcastle, DE, USA) at a heating rate of 20 °C/min ranging from 50 to 900 °C in 40 mL/min nitrogen flow with about 5 mg samples (powder) in air and nitrogen respectively. The measurements for DSC and TGA were repeated twice, and both of them showed similar results. Thermomechanical analysis (TMA) was performed on a TA instrument Q400 (TA, Newcastle, DE, USA) and the procedure was listed as follows: Heating the sample (20 mm (L) × 5 mm (W) × 15 μm (T)) to 250 °C at 5 °C/min in 50 mL/min nitrogen flow with and cooling down to 40 °C at 10 °C/min in 50 mL/min nitrogen flow, then reheating the sample to 400 °C at 5 °C/min in 50 mL/min nitrogen flow. The in-plane coefficient of thermal expansion (CTE) values of the polyimide films in the glassy state were calculated as an average from 50–200 °C in the second heating cycle of TMA. Ultraviolet-visible (UV–Vis) spectra were acquired using a PerkinElmer Lambda 650 UV–Vis spectrometer (PerkinElmer, Waltham, MA, USA) from 200 to 800 nm at a resolution of 2 nm. The mechanical properties of the polyimide films (25 mm (L) × 5 mm (W) × 15 μm (T)) were tested on an Instron universal tester 5567 (Instron, Canton, MA, USA) at ambient temperature at a crosshead speed of 6 mm/min.

## 3. Results and Discussion

### 3.1. Synthesis of Homopolyimide and Copolyimide

A series of homopolyimides and copolyimides were synthesized from TFMB diamine and two dianhydrides, 6FDA and HPMDA in DMAc with different HPMDA/6FDA mole ratios through a one-pot method. Table 1 summarizes the component and the GPC data of the copolyimides. These polymers exhibited reasonable molecular weight with *M*_n_ and *M*_w_ in the range of 2.71–4.51 × 10^4^, which was very important to ensure the fabrication of free-standing films. With the increasing content of alicyclic monomer (HPMDA), the molecular weight of the polyimide decreased due to the low reactivity of HPMDA compared to 6FDA. The proposed one-pot solution polycondensation method is considered suitable for polymerizing low reactive monomers as it simultaneously removes water evolved in imidization [7]. These results indicated that we had successfully synthesized copolyimides with moderate molecular weight through one-pot solution polycondensation in a relatively mild imidization temperature.

**Figure 1:**
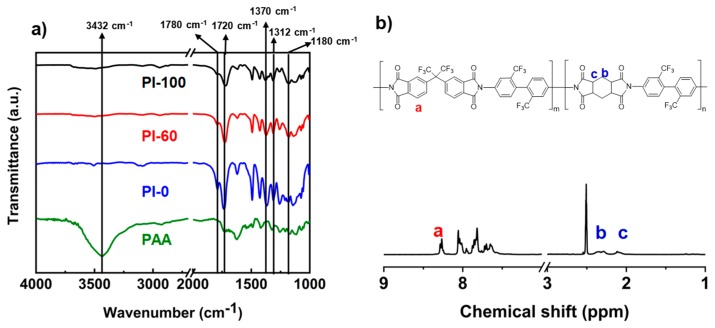
(**a**) FT-IR spectra of PI-100, PI-60, PI-0 and PAA. (**b**) ^1^H NMR spectrum of PI-40.

The structures of the polyimides were characterized by using FT-IR and ^1^H NMR spectra (Figure 1). The characteristic absorptions of imide groups were observed at about 1780 cm^−1^ (C=O, symmetric), 1720 cm^−1^ (C=O, asymmetric) and 1370 cm^−1^ (C–N, asymmetric) in the FT-IR spectra of PI-0, PI-60 and PI-100. The absorptions assigned to the C–F were detected at 1312 and 1180 cm^−1^. The characteristic N–H stretching vibrations in PAA ranging from 3300–3500 cm^−1^ disappeared in the FT-IR of PI, indicating that the PI was fully imidized. In the ^1^H NMR spectrum of PI-40, the chemical shift of 8.27 ppm and 2–2.43 ppm was assigned as the protons of aromatic ring (a) of 6FDA and alicyclic protons (b and c) of HPMDA, indicating that 6FDA and HPMDA had been successfully copolymerized in the PI chain. The chemical shift between 7.5–8.1 ppm was not well-separated due to the complex chain structure of copolyimides and assigned as other protons of aromatic ring of 6FDA and TFMB. As we can see in Figure S1, with the increase content of HPMDA, the intensity of alicyclic protons (b and c) increased and the intensity of aromatic protons (a) decreased. The results of ^1^H NMR showed we had successfully obtained copolyimides with different mole ratio of HPMDA/6FDA, which can be calculated from the following equation.

(1) $$M_{HPMDA}/M_{6FDA}=\frac{I_{2.43-2}}{4\;\times \;I_{8.27}}.$$

In the equation, *I*_2.43−2_ and *I*_8.27_ represents the integral, which corresponds to the peak whose chemical shift is between 2–2.43 ppm and 8.27 ppm respectively. The calculated mole ratios of HPMDA/6FDA in copolyimides PI-10, PI-40, PI-60 and PI-80 were 0.7:9.3, 2.8:7.2, 4.6:5.4 and 6.9:3.1 respectively. The reason why the calculated mole ratio of HPMDA/6FDA was lower than the feed ratio was attributed to the low reactivity of HPMDA resulted from its self-steric hindrance during polymerization [20].

### 3.2. Solubility

The solubility behavior of the polyimides was measured by dissolving polyimide powders at a concentration of 5 wt % in different organic solvents at room or elevated temperature. As shown in Table 2, all the copolyimides can be easily dissolved in most of the solvents, such as DMAc, DMF, DMSO, THF, EA, acetone and DCM. PI-0, PI-10 and PI-40 were partially dissolved in *m*-cresol, in contrast, PI-60, PI-80 and PI-100 could be dissolved in *m*-cresol at elevated temperatures. Most aromatic PIs have poor solubility and thus their processing on the industrial scale is difficult. In contrast, the good solubility of the copolyimide films might be useful in resolving the processing problem and extending the application value of copolyimides.

There are two main factors that influence the polymer solubility: Backbone rigidity and intermolecular interactions [3,35]. The copolyimides possessed good solubility because of the following reasons: First, the CF_3_ groups had large free volume and strong electronegativity and thus loosened the PI chain stacking, which was advantageous for reducing the intermolecular CT interactions of the polyimide chain [36]. Second, alicyclic structures provided by HPMDA enhanced the solubility of the copolyimides due to the inhibition of the inter- and intra-molecular CT interactions, which was ascribed to its relatively low molecular density and polarity [3].

### 3.3. Thermal Behavior

**Figure 2:**
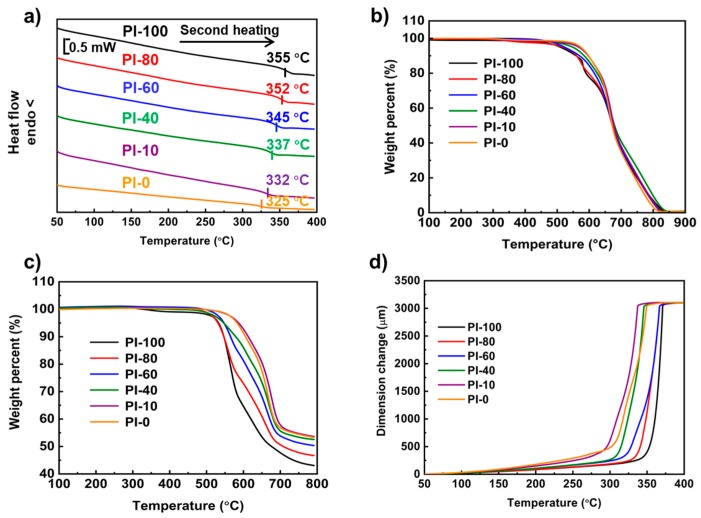
(**a**) Differential scanning calorimetry (DSC), (**b**) TGA in air, (**c**) TGA in N_2_ and (**d**) thermomechanical analysis (TMA) curves of homopolyimides and copolyimides.

In the present study, copolymerization was used to achieve the desired PIs with tunable thermal properties. The thermal properties of the polyimides were evaluated by DSC, TGA and TMA. The glass transition temperature of the polyimides with variable HPMDA contents was determined by DSC (Figure 2a and Table 3). The results showed considerable enhancement in *T*_g_ from 325 to 355 °C as HPMDA/6FDA ratio increased. Due to the large free volume presented by CF_3_ groups, PI-0 containing the highest content of CF_3_ exhibited the lowest *T*_g_ compared with other polyimides. As the content of CF_3_ groups reduced, the free volume between the polyimide chain decreased, resulting in the improvement of *T*_g_. On the other side, the molecular shape of the HPMDA-based diimide moieties unit (just like a “dish”) is more suitable for stacking [20]. As a result, *T*_g_ of the copolyimides was promoted with the increase content of HPMDA.

The thermogravimetric analysis of the copolyimides is shown in Figure 2b, Figure 2c and the results are listed in Table 3. Except for PI-100 (HPMDA/TFMB), all the copolyimides exhibited good thermal stability with the 5% and 10% weight decomposition temperature in the range of 517–567 and 560–596 °C in air, 531–586 and 547–613 °C in nitrogen respectively. The reason why the *T*_d_ value decreased as the concentration of HPMDA increased was explained as follows. With the increasing content of alicyclic dianhydride (HPMDA), the content of aromatic structure in the polyimide was decreased simultaneously. Compared with the aromatic structure, the alicyclic structure is easier to decompose under the condition of heating. As a result, the *T*_d_ value decreased as the concentration of HPMDA increased. The DTG curves of PIs in air and N_2_ had differences and similarities as shown in Figure S2. Two peaks in the range of 500–600 and 600–700 °C representing the degradation of alicyclic and aromatic structure respectively, existed in both DTG curves. With the increasing content of HPMDA, the intensity of the peak in 600–700 °C decreased and that of the peak in 500–600 °C increased. However, when the samples were heated to 700–800 °C, the decomposition rate still kept around 0.3%/°C in air while the decomposition almost stopped in N_2_. The difference between the DTG curves in air and N_2_ was mainly attributed to the existence of oxygen in air, resulting in different thermal degradation mechanism, which required further investigation.

The thermal dimensional stability of the PI films was represented by the coefficient of thermal expansion (CTE). The CTE values showed obvious decline after the introduction of HPMDA (Figure 2d and Table 3). PI-100 decreased by approximately 54% compared with that of PI-0 (148.9 ppm/K) and reached 68.7 ppm/K. This improvement was attributed to the rigid structure provided by HPMDA. Compared with PI-0, thermal studies clearly indicated that the incorporation of HPMDA in the polyimides enhanced *T*_g_ and reduced CTE, however the thermal stability changed significantly.

### 3.4. Optical Properties

**Figure 3:**
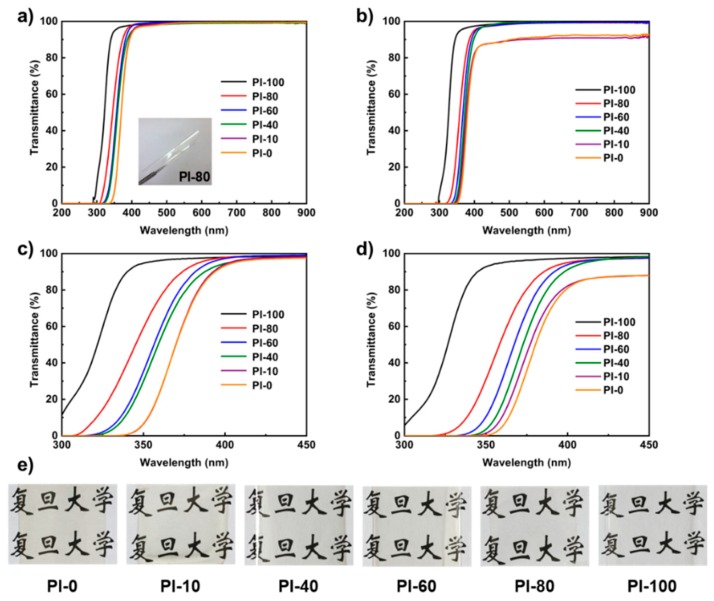
(**a**) UV–Vis spectra of homopolyimide and copolyimide films and photograph of PI-80 film with a thickness of about 15 μm. (**b**) UV–Vis spectra of homopolyimide and copolyimide films with a thickness of about 45 μm. (**c**) UV–Vis spectra of homopolyimide and copolyimide films with a thickness of about 15 μm in 300–450 nm. (**d**) UV–Vis spectra of homopolyimide and copolyimide films with a thickness of about 45 μm in 300–450 nm. (**e**) Photographs of homopolyimide and copolyimide films with a thickness of about 45 μm. The films were placed on paper with the Chinese name of Fudan University to show the optical transparency.

Figure 3 presents the photographs and UV–Vis spectra of the copolyimide films with thickness about 15 and 45 μm. It was noted that these films were transparent and almost colorless. All films with a thickness of about 15 μm possessed excellent optical transmittance (*T*_400_ > 90% and *T*_500_ > 95%) in the visible region and a low cutoff wavelength (*λ*_0_) ranging from 289–333 nm (Table 4). Following the film thickness increased to 45 μm, the optical transmittance had a slight decrease, while the films still kept colorless in the visible region (*T*_400_ > 80%, *T*_500_ > 90% and *λ*_0_ ~297–346 nm). As the HPMDA loading increased, the transmittance of the polyimide films increased and the color gradually became pale. As we know, a colorless PI film should meet the requirements of *T*_400_ ≥ 80% with a thickness of 20 μm [7]. The copolyimide films exhibited high optical transparency around 400 nm (*T*_400_ > 80%) with a thickness of 45 μm and completely satisfied the requirements, thus it can be classified as CPI. It was worth mentioning that, the PI-100 films were somewhat brittle after being peeled off from the substrate in many cases, while 6FDA-contained films showed better quality of film as shown in the insert of Figure 3a.

The copolyimide films possessed good transmittance in the visible region owing to the following reasons. First, the PI chain stacking was loosened due to the existence of CF_3_ groups, which was advantageous for reducing inter-molecular CT interactions, resulting in decrease of film coloration and increase of transmittance [6,7]. Second, the alicyclic structure of HPMDA broke the conjugative effect of the main chain and inhibited CT interaction. Thus, the CT interactions were further reduced.

Here, the overall performance of the copolyimide films was compared with that of recently reported system. A series of semi-alicyclic PI were prepared from a hydrogenated 3,3’,4,4’-biphenyltetracarboxylic dianhydride and various aromatic diamines through a high-temperature polycondensation procedure. The semi-alicyclic PIs exhibited *T*_g_ in the range of 171.5–296.3 °C and *T*_d_^5^ neared 470 °C, and the transmittance at 450 nm was lower than 90% [37]. HPMDA was polymerized with an aromatic diamine to obtain polyimides using chemical imidization at low temperatures (40–160 °C). Owing to the low imidization temperature and alicyclic structure, the polyimide possessed good optical transmittance, which was higher than 90% at 400 nm, while the *T*_g_ was lower than 310 °C [38]. In this work, the copolyimide films displayed high thermal stability (*T*_g_ ~332–352 °C) due to the chain structure of the PI, and good optical properties (*λ*_0_ ~305–333 nm and *T*_400_ > 90%) attributed to weak CT interactions.

### 3.5. Mechanical Properties

The mechanical properties of the polyimide films are listed in Table 5. Tensile strength, elongation at break and elastic modulus were determined as averages of five drawing experiments. All these films exhibited appreciable mechanical stiffness, tensile strength varying from 91 to 167 MPa, elongation at break from 41% to 56% and tensile modulus in the range of 501–788 MPa. The copolyimides can be processed into transparent, free standing and tough films, indicating they possessed good mechanical properties.

There are many factors that affect the mechanical properties of polyimide, such as chemical structure, aggregation structure, molecular weight, preparing technique, thermal history and test conditions. In this study, the difference of the mechanical properties was not very obvious to make a relationship with the composition of PI. At this moment, the authors do not have a good reason to explain why the PI-10 and PI-40 films possess higher tensile strength and elastic modulus. It seems to be mainly related to the intermolecular interaction and molecular aggregation, which requires further investigation.

## 4. Conclusions

A series of CPI films with different mole ratio of HPMDA/6FDA were successfully obtained by copolymerizing of TFMB, 6FDA and HPMDA through one-pot solution polycondensation and the relationship between the polymer components and their properties was investigated. Incorporation of alicyclic dianhydride into fluorinated polyimide improves the optical transparency and *T*_g_ of the resulting polyimides. Meanwhile, the drawback of low decomposition temperature of alicyclic polyimide was compensated with increasing fluorinated dianhydride content. These colorless copolyimide films possess good solubility in most organic solvents, high thermal stability (*T*_g_ ~332–352 °C) and excellent optical properties (*T*_400_ > 90% and *λ*_0_ ~305–333 nm). The existence of CF_3_ groups and alicyclic structure loosens the PI chain stacking and breaks the conjugative effect of the main chain, thereby reducing the inter- and intra-molecular CT interactions, which results in a decrease of film coloration and increase of solubility. Among the obtained polyimides, PI-40 shows best overall performance with high thermal stability and transmittance. In summary, this work provides new insights on preparing CPI films with a combination of good solubility, high thermal stability and excellent optical properties, which exhibits great potential in the applications of substrates for optoelectronic engineering.

## Figures and Tables

**Table 1:** Monomer component and gel permeation chromatography (GPC) data of homopolyimides and copolyimides.

Sample	HPMDA/6FDA (mmol/mmol)	TFMB (mmol) ^a^	*M*_n_ (×10^4^ g/mol)	*M*_w_ (×10^4^ g/mol)	PDI
PI-0	0:10	10	4.51	6.63	1.47
PI-10	1:9	10	4.51	7.36	1.63
PI-40	4:6	10	3.43	6.05	1.76
PI-60	6:4	10	3.68	6.10	1.66
PI-80	8:2	10	3.14	5.74	1.82
PI-100	10:0	10	2.71	4.78	1.76

^a^ The mole ratio of diamine (TFMB) to the two dianhydrides (HPMDA and 6FDA) was 1:1.

**Table 2:** Solubility of homopolyimides and copolyimides. ^a^

Sample	DMAc	DMF	DMSO	THF	EA	Acetone	DCM	*m*-Cresol	MeOH
PI-0	++	++	++	++	++	++	++	+-	-
PI-10	++	++	++	++	++	++	++	+-	-
PI-40	++	++	++	++	++	++	++	+-	-
PI-60	++	++	++	++	++	++	++	+	-
PI-80	++	++	++	++	++	++	++	+	-
PI-100	++	++	++	++	++	++	++	+	-

^a^ “++” represents soluble at room temperature. “+” represents soluble upon heating. “+-” represents partially soluble. “-” represents insoluble even on heating.

**Table 3:** Thermal properties of homopolyimides and copolyimides.

Sample	*T*_g_ (°C) ^a^	In Air		In Nitrogen		CTE (ppm/K) ^f^
		*T*_d_^5^ (°C) ^b^	*T*_d_^10^ (°C) ^c^	*T*_d_^5^ (°C) ^d^	*T*_d_^10^ (°C) ^e^	
PI-0	325	573	597	582	606	148.9
PI-10	332	567	596	586	613	123.7
PI-40	337	547	584	546	580	97.0
PI-60	345	523	569	547	565	87.3
PI-80	352	517	560	531	547	75.6
PI-100	355	491	551	532	547	68.7

^a^*T*_g_ was measured by DSC. ^b^ The 5% weight decomposition temperature was measured by TGA in air. ^c^ The 10% weight decomposition temperature was measured by TGA in air. ^d^ The 5% weight decomposition temperature was measured by TGA in N_2_. ^e^ The 10% weight decomposition temperature was measured by TGA in N_2_. ^f^ The coefficient of thermal expansion was measured by TMA.

**Table 4:** Optical properties of homopolyimide and copolyimide films with thickness of about 15 and 45 μm.

Sample	15 μm Film			45 μm Film		
	*λ*_0_ (nm) ^a^	*T*_400_ (%) ^b^	*T*_500_ (%) ^c^	*λ*_0_ (nm)	*T*_400_ (%)	*T*_500_ (%)
PI-0	333	93.3	98.5	346	81.0	90.1
PI-10	333	94.1	99.0	340	82.2	90.5
PI-40	318	94.6	98.4	339	91.2	99.2
PI-60	313	97.3	99.5	331	93.9	98.4
PI-80	305	97.9	99.4	315	95.1	98.4
PI-100	289	98.0	99.2	297	97.4	98.9

^a^ Cutoff wavelength. ^b^ Optical transmittance at 400 nm. ^c^ Optical transmittance at 500 nm.

**Table 5:** Mechanical properties of homopolyimide and copolyimide films.

Sample	Tensile Strength (MPa)	Elongation at Break (%)	Elastic Modulus (MPa)
PI-0	91.4 ± 3.0	41.9 ± 2.7	501.1 ± 52.8
PI-10	167.3 ± 5.4	55.9 ± 3.7	702.9 ± 25.9
PI-40	155.1 ± 26.8	40.9 ± 2.8	787.7 ± 111.9
PI-60	126.3 ± 5.4	48.5 ± 3.7	595.3 ± 42.1
PI-80	119.6 ± 13.0	48.1 ± 3.0	546.2 ± 60.0
PI-100	122.2 ± 10.8	54.2 ± 3.3	534.0 ± 18.5

## Supplementary Materials

The following are available online at https://www.mdpi.com/2073-4360/11/8/1319/s1, Figure S1: ^1^H NMR spectra of PI-100, PI-80, PI-60, PI-10 and PI-0, Figure S2: The derivative thermal gravity (DTG) curves of homopolyimides and copolyimides in air (**a**) and N_2_ (**b**).
