# Supplementary material for: Colorless Semi-Alicyclic Copolyimides with High Thermal Stability and Solubility
Source: Polymers (Basel). 2019 Aug 7;11(8):1319. doi: 10.3390/polym11081319 (PMC6723692; doi:10.3390/polym11081319)
Supplement: Supplementary file 1 [file polymers-11-01319-s001.pdf]

Supporting Information

Colorless Semi-Alicyclic Copolyimides with High Thermal Stability and Solubility

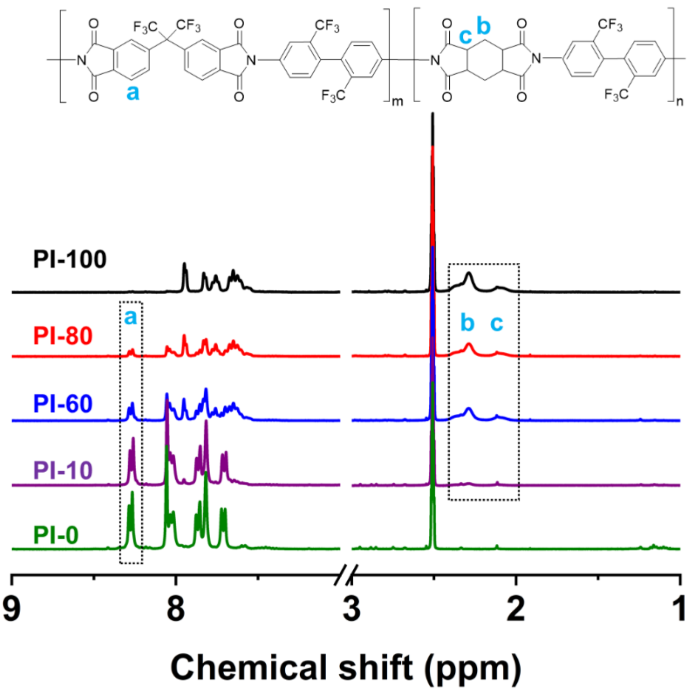

Figure S1. ^{1}H NMR spectra of PI-100, PI-80, PI-60, PI-10 and PI-0.

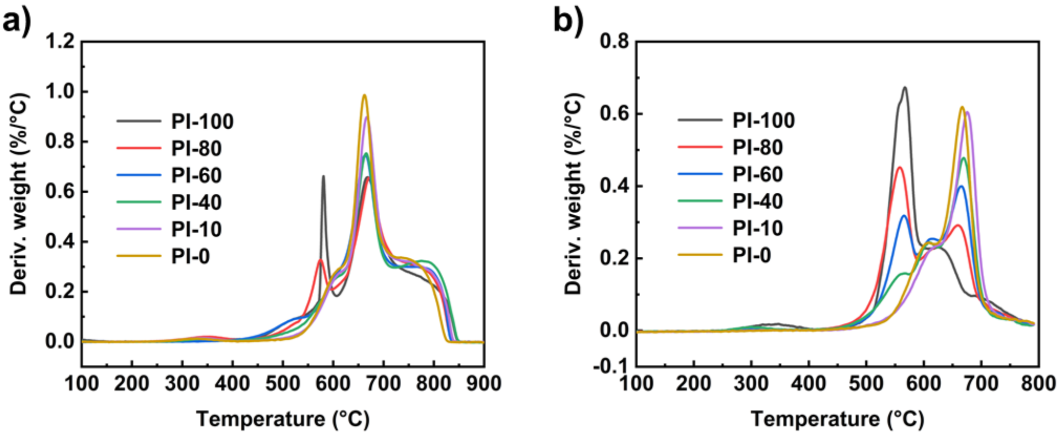

Figure S2. The derivative thermal gravity (DTG) curves of homopolyimides and copolyimides in air (a) and N_2 (b).
